# Cytotoxic Potential of *Bacillus cereus* Strains ATCC 11778 and 14579 Against Human Lung Epithelial Cells Under Microaerobic Growth Conditions

**DOI:** 10.3389/fmicb.2016.00069

**Published:** 2016-02-03

**Authors:** Kathleen Kilcullen, Allison Teunis, Taissia G. Popova, Serguei G. Popov

**Affiliations:** School of Systems Biology, George Mason UniversityManassas, VA, USA

**Keywords:** *Bacillus cereus*, culture filtrates, cytotoxicity, lung epithelial cells, cereolysin O

## Abstract

*Bacillus cereus*, a food poisoning bacterium closely related to *Bacillus anthracis*, secretes a multitude of virulence factors including enterotoxins, hemolysins, and phospholipases. However, the majority of the *in vitro* experiments evaluating the cytotoxic potential of *B. cereus* were carried out in the conditions of aeration, and the impact of the oxygen limitation in conditions encountered by the microbe in natural environment such as gastrointestinal tract remains poorly understood. This research reports comparative analysis of ATCC strains 11778 (BC1) and 14579 (BC2) in aerobic and microaerobic (static) cultures with regard to their toxicity for human lung epithelial cells. We showed that BC1 increased its toxicity upon oxygen limitation while BC2 was highly cytotoxic in both growth conditions. The combined effect of the pore-forming, cholesterol-dependent hemolysin, cereolysin O (CLO), and metabolic product(s) such as succinate produced in microaerobic conditions provided substantial contribution to the toxicity of BC1 but not BC2 which relied mainly on other toxins. This mechanism is shared between CB1 and *B. anthracis*. It involves the permeabilization of the cell membrane which facilitates transport of toxic bacterial metabolites into the cell. The toxicity of BC1 was potentiated in the presence of bovine serum albumin which appeared to serve as reservoir for bacteria-derived nitric oxide participating in the downstream production of reactive oxidizing species with the properties of peroxynitrite. In agreement with this the BC1 cultures demonstrated the increased oxidation of the indicator dye Amplex Red catalyzed by peroxidase as well as the increased toxicity in the presence of externally added ascorbic acid.

## Introduction

*Bacillus cereus* is a Gram-positive spore-forming bacterium widely present in the environment. It is a soil saprophyte that can adapt and proliferate in the lower sections of the human gastrointestinal tract. It is also an opportunistic pathogen responsible for local and systemic infections as well as food poisoning of an emetic or diarrheal type (Stenfors Arnesen et al., 2008). *B. cereus* secretes a multitude of pathogenic factors that were suggested to contribute synergistically toward its toxicity. These factors reflecting diverse lifestyles of the microbe include metalloproteases, collagenase, phospholipases, emetic toxin, enterotoxins, and hemolysins (Gohar et al., 2005). The diarrheal syndrome is attributed to enterotoxins: hemolysin BL (Hbl), non-hemolytic enterotoxin (Nhe), and cytotoxin K (CytK). These pore-forming toxins (PFTs) disrupt the membrane of epithelial cells lining the gastrointestinal tract (Senesi and Ghelardi, 2010). Other enterotoxins: FM (EntFM), S (EntS), and T (BceT) may also contribute to the pathogenicity (Asano et al., 1997; Lund et al., 2000; Hansen and Hendriksen, 2001; Fagerlund et al., 2004; Kim et al., 2015). *B. cereus* secretes additional two beta-barrel pore-forming hemolysins, cereolysin O (CLO) and hemolysin II (HlyII), that are non-diarrheal (Bernheimer and Grushoff, 1967; Andreeva et al., 2006; Ramarao and Sanchis, 2013). Virulence of the emetic strains is related to cereulide, a thermostable cyclic dodecadepsipeptide synthesized by a non-ribosomal peptide synthetase encoded by *ces* genes (Ehling-Schulz et al., 2005, 2006). Products from other genes such as hemolysin A (*hlyA*), hemolysin III (*hlyIII*), phosphatidylinositol-specific phospholipase C (*plcA*), cereolysin A or phospholipase C (*cerA*), cereolysin B or sphingomyelinase (*cerB*), and Immune inhibitor A (*InhA*) are also involved in the pathogenesis of *B. cereus* (Baida and Kuzmin, 1995; Schoeni and Lee Wong, 2005; Hendriksen et al., 2006; Stenfors Arnesen et al., 2008; Oda et al., 2012; Doll et al., 2013).

The biological significance of the above factors in the context of their contribution to bacterial virulence and persistence in the particular environmental conditions is not completely understood. A closely related human pathogen *Bacillus anthracis* adopted a different strategy with a lesser number of pathogenic factors. Both microbes were previously considered the same species (Helgason et al., 2000). DNA sequencing of the *B. anthracis* and *B. cereus* type strains (Ivanova et al., 2003) confirmed high similarity or their genomes, but revealed a number of important differences. In contrast to the majority of *B. cereus* strains, the higher pathogenicity of *B. anthracis* seems to rely on the contribution of the plasmid-born Lethal and Edema Toxins and the poly-γ-D-glutamic acid capsule. However, this distinction is not absolute, and recent studies identified *B. cereus* pathogenic isolates expressing homologs of Lethal Toxin and polysaccharide capsules functionally similar to the poly-γ-D-glutamic one (Hoffmaster et al., 2004, 2006). On the other hand, *B. anthracis*’s potency to produce hemolytic factors is considerably lower compared to *B. cereus*. The former does not contain the enterotoxins (except Nhe reported by Mendelson et al., 2004) and the activity of the CLO analog, anthrolysin O (ALO), is restricted to the anaerobic conditions (Klichko et al., 2003). However, the activity of ALO seems to be important for *B. anthracis*. Experiments *in vivo* with ALO-null mutants revealed substantial contribution of ALO together with phospholipases to the virulence (Heffernan et al., 2007). Cowan et al. (2007) reported high toxicity of intravenously administered ALO, and Nakouzi et al. (2008) demonstrated a protective effect of monoclonal antibodies against ALO in mice challenged intravenously with vegetative bacteria.

We previously reported a novel mechanism of *B. anthracis* metabolic toxicity mediated by ALO and succinate as a fermentation product produced by bacteria in the conditions of reduced oxygen availability (Popova et al., 2011). The combined effect of ALO and succinate results in the permeabilization of the cell membrane and oxidative stress in the exposed lung epithelial cells. In addition, it was discovered that the presence of bovine serum albumin (BSA) potentiates the toxicity of the *B. anthracis* (Popova et al., 2011; St John et al., 2013). It was suggested that BSA could concentrate in its hydrophobic core the nitric oxide (NO) produced by bacteria, followed by the micellar catalysis of NO conversion into reactive species such as peroxynitrite in the host cells under oxidative stress.

In this study, using two *B. cereus* strains expressing different sets of pathogenic factors (**Table 1**) we wanted to determine to what extent these mechanisms of *B. anthracis* toxicity are relevant to *B. cereus*. Specifically we were interested in evaluating the contribution of CLO to the host cell membrane damage relative to other PFTs as well as the impact of reduced oxygen availability on *B. cereus* toxicity. Most of the previous *B. cereus* toxicity studies were conducted in aerobic conditions and therefore did not reflect significant changes in the levels of pathogenic factors which can take place in response to redox state of the environment. Since *B. cereus* infections typically occur in anoxic or hypoxic conditions such as those in a gastrointestinal tract, it adapts its metabolism and regulates its proteome in response to changes in oxygen pressure (Ouhib-Jacobs et al., 2009; Clair et al., 2010; Laouami et al., 2014). In connection with the role of reactive nitrogen species generated by *B. anthracis* and reports on release of peroxynitrite by *B. cereus* in microaerobic cultures (Mols et al., 2010; Mols and Abee, 2011) we also investigated the effect of BSA on the toxicity of *B. cereus*. We report a novel mechanism involving synergistic activity of the pore-forming toxin and metabolic products of *B. cereus* enhanced by serum albumin in microaerobic conditions.

**Table 1:** Virulence factors in *B. cereus* ATCC 11778 and 14579.

Virulence factor	Size (kDa)	ATCC strain	
		11778 (BC1)	14579 (BC2)
Immune inhibitor A (InhA)	87.9	+	+
Phospholipase C (PLC)	23.0	+	+
Sphingomyelinase (SMase)	34.0	+	+
Cereolysin AB (CerAB)	67.0	+	+
Collagenase	109.0	+	+
Cereulide	1.2	–	–
Hemolysin BL (Hbl)	45.0 (L2)	–	+
	36.0 (L1)		
	35.0 (B)		
Non-hemolysin E (Nhe)	41.0 (A)	+	+
	39.8 (B)		
	36.5 (C)		
Cytotoxin K (CytK, HlyIV)	34.0	–	+
Cereolysin O (CLO, HlyI)	52.5	+	+
Hemolysin II (HlyII)	45.6	+	+
Hemolysin III (HlyIII)	24.4	+	+

*+/– Denotes whether gene is present in strain (Hansen and Hendriksen, 2001; Gohar et al., 2005; Kumar et al., 2010; Kim et al., 2015).*

## Materials and Methods

### Reagents

All reagents used were from Sigma–Aldrich unless specified otherwise. Cholesterol was dissolved in ethanol at 1 mg mL^-1^and then further diluted into bacterial culture supernatants (Sups). Ham’s F-12 cell culture medium and Complete Serum-Free Medium (CSFM) were from Mediatech Inc., Manassas, VA, USA. Formulated Dulbecco’s Modified Eagle Medium (DMEM) came from Sigma–Aldrich. BSA was of >98% purity and essentially free from globulins and fatty acids. Amplex Red (AR) dye was from Invitrogen. The CytoTox-ONE Homogeneous Membrane Integrity Assay came from Promega, Madison, WI, USA. Succinic acid (SA) concentration was measured using SA assay kit from Megazyme. Protein concentration was estimated using the Bradford protein assay from Bio-Rad. Rabbit anti-streptolysin O antibody was from B-Bridge International, Santa Clara, CA, USA. Anti-rabbit IgG, HRP-linked antibody came from Cell Signaling Technology.

### Assay Kits

The listed kits were performed according to the manufacturers’ protocols unless otherwise indicated. Cell permeability was measured using the CytoTox-ONE Homogeneous Membrane Integrity Assay which indicated the amount of lactate dehydrogenase (LDH) secreted from cells with a damaged membrane. LDH was measured with a 10-minute coupled enzymatic assay that results in the conversion of resazurin into fluorescent resorufin detected at 530/590 nm. The extent of permeability was calculated as fluorescence relative to completely lysed cells.

For determining the presence of hydrogen peroxide and peroxynitrite the dye AR from Invitrogen was used with a modified protocol. AR (*N*-acetyl-3,7-dihydroxyphenoxazine) is a colorless derivative of dihydroresorufin that when oxidized produces a colorful fluorescent product resorufin which is detectable at 571 nm. AR and horseradish peroxidase (HRP) were added to DMEM without Phenol Red indicator supplemented with 1 g L^-1^ of BSA at a concentration of 0.1 mM and 0.2 U mL^-1^, respectively, prior to inoculation with bacteria as described below. Cultures were collected every hour, centrifuged at 10,000 × *g* for 5 min, and the Sups were removed. The absorbance of Sups was read at 571 nm *via* spectrophotometer.

### Bacterial Strains, Culture Conditions, and Preparation of Culture Sups

The following procedure for bacterial propagation was used for every experiment unless stated otherwise. *B. cereus* strains 11778 and 14579 (designated by us as BC1 and BC2, respectively) were from the ATCC collection (Manassas, VA, USA). They were grown on agar plates containing Luria broth. Agar plates were re-streaked every seven days and were kept at 8°C until inoculation. Single colonies were used to inoculate into Luria broth and cultures were kept in an incubator shaker at 200 rpm, 37°C for 18 h. Overnight culture at 1:100 dilution was inoculated into either 10 mL of Complete Serum-Free Medium (CSFM), a nitrate rich medium containing 1 g L^-1^ of BSA, or DMEM supplemented with 1 g L^-1^of BSA. Cultures were grown either under microaerobic (stationary) conditions, in a 6-well plate in an incubator at 37°C, 5% CO_2_, or under aerobic conditions, in loosely capped 50 mL tube shaken at 200 rpm at 37°C for 20 h unless specified otherwise. Under stationary conditions, the bacteria consume available oxygen and gradually become hypoxic, thus representing a microaerobic environment. Cultures were collected and the optical density (OD) of 200 μL of bacterial suspensions in a 96-well plate were measured in triplicates using a microplate reader at 600 nm.

Bacterial suspensions were centrifuged at 3000 × *g* for 15 min and Sups were removed from the bacterial pellet. Penicillin (100 μg mL^-1^) and streptomycin (100 U mL^-1^) were added to Sups to prevent any bacterial contamination. Fresh Sups were used immediately for challenge experiments.

### Cell Cultivation and Toxicity Studies

Human small-airway epithelial cells (primary HSAECs) were from Cambrex Inc., Walkersville, MD, USA. Cells were cultured in Ham’s F-12 medium containing 10% fetal bovine serum, non-essential amino acids, L-glutamine, and pyruvate and grown in 37°C in a 5% CO_2_ atmosphere. Cells were seeded into a 96-well plate at a density of 2.5 × 10^4^ per well and grown to confluence. For cytotoxicity assays, 200 μl of bacterial culture Sups were added per well and incubated for 20 min at 37°C, 5% CO_2_ without shaking. After cell exposure, the plate was spun at 2000 × *g* for 5 min, Sups were removed, and 200 μL of 5% resazurin, a redox dye, dissolved in CSFM was added to each well. Resazurin measures cell survival because cellular metabolism breaks down the dye changing its color and fluorescence. Fluorescence was measured with an excitation at 530 nm and emission at 590 nm *via* a fluorescence reader to determine the differences in cell viability after 2-h incubation with resazurin. Viability was calculated as fluorescence relative to mock-treated cells. Overall, the assay generated consistent results; however, some variability in Sup toxicity was noticed between independently grown HSAEC cultures. This effect was not studied further.

### Sup Fractionation

For size exclusion chromatography experiments, bacteria were cultured in DMEM with or without BSA (1 g L^-1^) in 6-well plates under microaerobic conditions at 37°C, 5% CO_2_ for 20 h. Sups were collected and concentrated eightfold, *via* SpeedVac at 32°C. 1 mL of Sup concentrate was injected into a HiPrep 16/60 Sephacryl S-200 HR column (GE Healthcare Life Sciences) and run at a rate of 4 mL min^-1^ in a 50 mM Tris running buffer, pH 7.5. Flow-through fractions were collected every 2 min between a run time of 13 and 135 min. The toxicity of fractions to HSAECs was assessed immediately using the 100 μL fractions which were added to a 96-well plate of confluent HSAECs containing 100 μL of DMEM per well. Cells were exposed for 1 h and resazurin was used as an indicator for cell survival. The fractions were stored at –20°C for mass spectrometry (MS) analysis.

### MS Analysis

To determine the protein composition of fractions, samples were prepared for liquid chromatography-tandem mass spectrometry (LC-MS/MS). Fractions were first concentrated 10-fold *via* SpeedVac at 32°C. Samples were resuspended and reduced in an 8 M urea/10 mM DTT mix for 30 min, alkylated by 50 mM iodoacetamide in the dark for 30 min, and then finally digested by trypsin (Thermo Fisher Scientific) at 37°C overnight. Peptides were purified *via* Zip-Tip (Millipore) and samples were analyzed by LC-MS/MS using a linear ion-trap mass spectrometer (LTQ, Orbitrap). After sample injection, the column was washed for 5 min with mobile phase A (0.4% acetic acid) and peptides eluted using a linear gradient of 0% mobile phase B (0.4% acetic acid, 80% acetonitrile) to 50% mobile phase B in 30 min at 250 nL/min, then to 100% mobile phase B for an additional 5 min. The LTQ mass spectrometer was operated in a data-dependent mode in which each full MS scan was followed by five MS/MS scans where the five most abundant molecular ions were dynamically selected for collision-induced dissociation using normalized collision energy of 35%. Tandem mass spectra were collected by Xcalibur 2.0.2 and searched against the NCBI mouse protein database using SEQUEST (Bioworks 3.3.1 software from Thermo Fisher) using tryptic cleavage constraints. Mass tolerance for precursor ions was 5 ppm and mass tolerance for fragment ions was 0.25 Da. SEQUEST filter criteria were: Xcorr *vs*. charge 1.9, 2.2, 3.5 for 1+, 2+, 3+ ions; maximum probability of randomized identification of peptide <0.01.

### SDS-PAGE, Native PAGE, and Western Blotting

For native PAGE and SDS-PAGE, protein fractions were mixed with a DNA-loading buffer for native PAGE or with 2x Laemmli sample buffer supplemented with 50 mM DTT for SDS-PAGE. Fractions for SDS-PAGE were boiled with the buffer for 5 min. Samples were separated using 4–20% Tris-Glycine gels and then transferred to nitrocellulose membranes using iBlot Dry Blotting System (Life Technologies). Membranes were blocked with 5% BSA in PBS with 0.01% Tween 20 (PBST) for 1 h, and then incubated in a rabbit anti-streptolysin O antibody (1:1000) overnight. Membranes were washed in PBS with 0.05% Tween-20 and then incubated with an anti-rabbit IgG, HRP-linked antibody (1:5000) for 1 h at room temperature. Western blots were developed using SuperSignal West Dura Extended Duration Substrate (Thermo Fisher Scientific) and then imaged using ChemiDoc XRS+ System (Bio-Rad).

### Statistical Analysis

Each measurement was done in triplicate and experiments were performed at least twice for consistency. In figures, error bars indicate 95% confidence intervals of mean (two-tail *t*-test).

## Results

### BC1 and BC2 Cultures Grown in Microaerobic and Aerobic Conditions Accumulate High Levels of Toxicity

**FIGURE 1:**
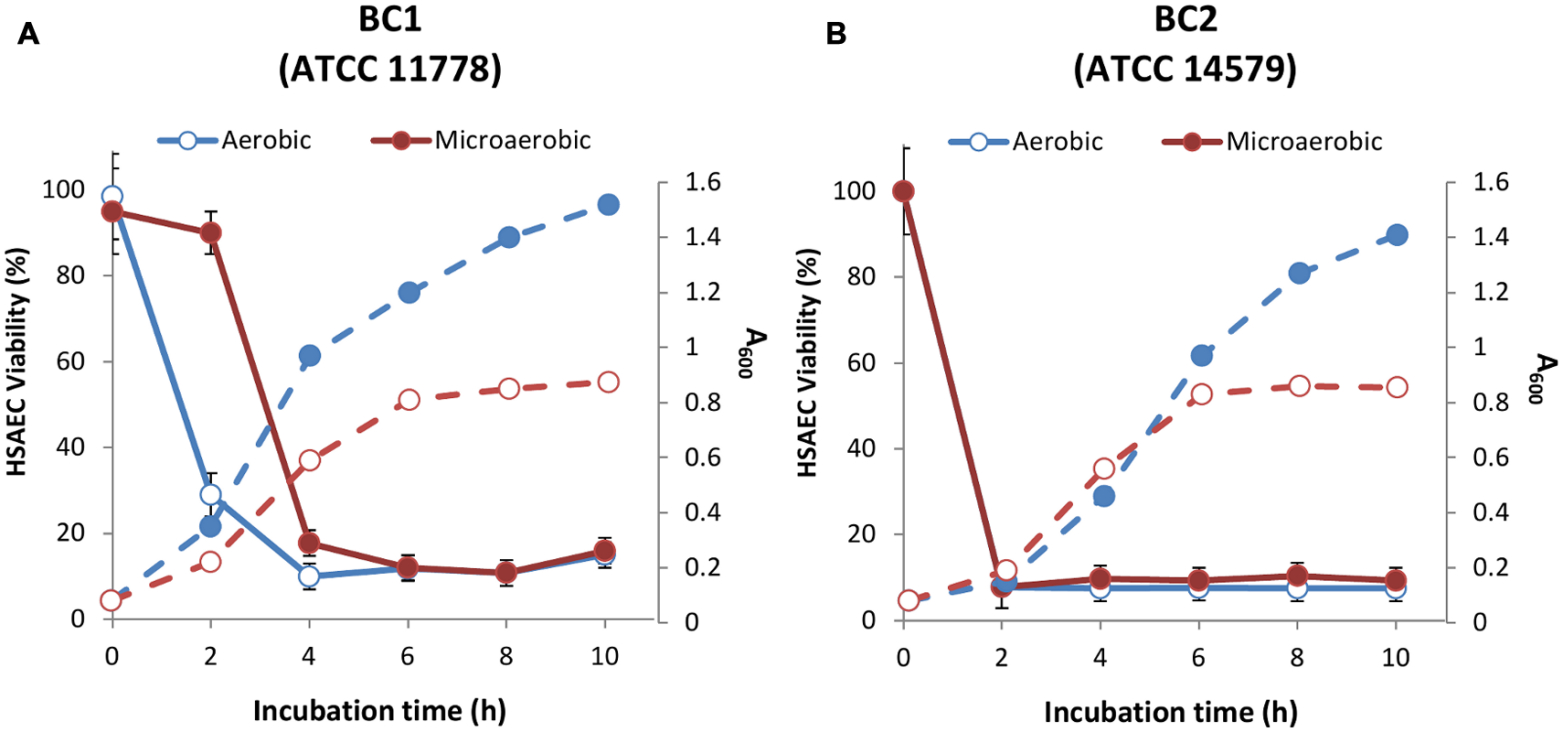
**The growth (dashed lines) and toxicity (solid lines) of BC1 (A) and BC2 (B) under microaerobic and aerobic conditions (filled and open circles, correspondingly).** Overnight cultures were grown in aerated LB medium and used to inoculate CSFM (1:100). For static microaerobic cultures the inoculated medium was dispensed into 24-well plate (2 mL per well) and incubated at 37°C and 5% CO_2_ without shaking. For aerobic cultures, 20 mL of inoculated CSFM in a 50 mL Falcon tube were shaken at 200 rpm and 37°C. The cultures were harvested every hour post-inoculation and their OD measured at 600 nm. Bacteria were pelleted and the Sups were used to expose HSAECs for 2 h at 37°C. Cell viability was assessed relative to untreated HSAECs using resazurin as described in the Section “Materials and Methods.”

**FIGURE 2:**
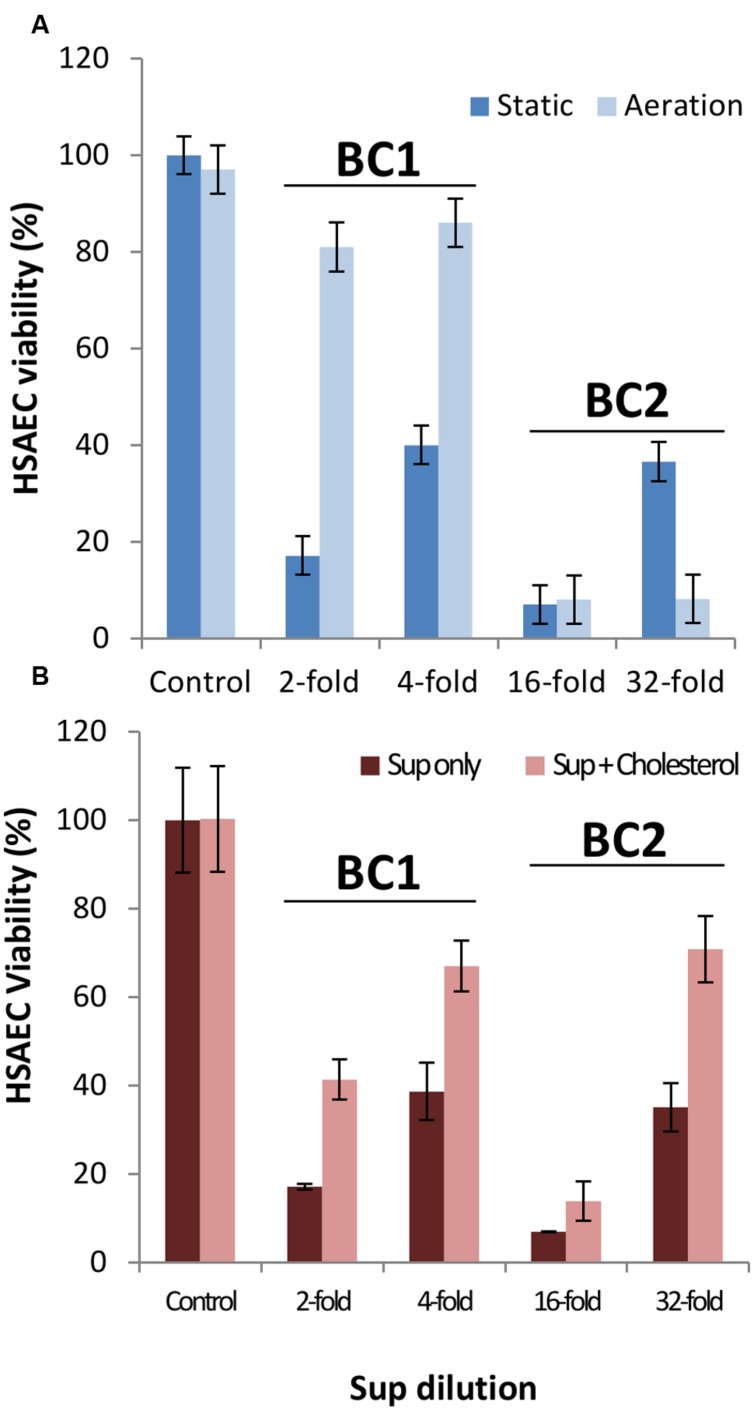
**BC1 and BC2 cultures accumulate high levels of toxicity when grown in microaerobic and aerobic conditions for 20 h (A).** The toxicity of microaerobic Sup can be partially abrogated by cholesterol **(B)**. **(A)** BC1 and BC2 were cultured in CSFM as described in the **Figure 1** legend. The Sups were collected, serially diluted in CSFM, and used to treat HSAECs for 20 min. The viability was assessed with resazurin relative to unexposed cells. **(B)** Where indicated, cholesterol was added to microaerobic Sups at 10 μg mL^-1^ for 1 h. For each strain the data for two dilutions in the dynamic range of the assay are shown.

To determine bacterial culture supernatant (Sup) toxicity toward HSAECs we used two *B. cereus* non-emetic strains, ATCC 11778 and 14579 (designated BC1 and BC2, respectively). The BC2 was reported to express full toxinogenic potential including enterotoxins Hbl, Nhe, CytK, BceT, phospholipases, and the pore-forming CLO (Gohar et al., 2005). BC1 does not express enterotoxins except Nhe (Hansen and Hendriksen, 2001; Gohar et al., 2005), and our preliminary data indicated that it produced CLO (not shown). We compared the acidification and toxicity of Sups grown in aerobic and microaerobic conditions. The aerobic culture was shaken to allow gas diffusion throughout the volume of the bacterial culture. In the microaerobic conditions the amount of oxygen available to bacteria sedimented to the bottom of the wells is strongly limited by diffusion. The onset of anaerobic fermentation was detectable as a reduction of culture pH approximately after 4-h incubation. After 10-h inoculation the pH of microaerobic Sups was close to 5.3, whereas aerobic Sups showed only a small shift from 7.0 to 6.8. Sups demonstrated a detectable cytotoxicity as early as 2 h post-inoculation corresponding to early exponential phase (**Figure 1**). The release of toxic factors continued upon further incubation. As shown in **Figure 2A**, the Sups of both strains after 20 h had to be diluted with fresh medium in order to be tested in the dynamic range of the resazurin cell viability assay. BC2 showed the highest toxicity in aerobic cultures killing almost all HSAECs at 32-fold dilution when the exposure time was reduced to 20 min from 2 h (**Figure 2**). Less toxic BC1 Sups showed higher activity in microaerobic conditions compared to aeration.

### Cholesterol Partially Inhibits the Permeabilization Activity and Toxicity of Microaerobic Sups Toward HSAECs

To reveal the contribution of CLO to the toxicity of BC1 and BC2 the Sups of microaerobic and aerobic cultures were incubated with 10 μg mL^-1^of cholesterol for 1 h prior to HSAEC exposure. CLO is a thiol-activated PFT that is known to be inhibited by the addition of cholesterol (Tweten, 2005). Results from **Figure 2B** demonstrate cholesterol caused a substantial reduction in the toxicity of microaerobic Sups from both strains. The incubation of cholesterol with Sups from aerobic cultures had little effect (data not shown) consistent with downregulation of CLO expression or/and its inactivation by oxygen (Tweten, 2005). The partial abrogation of Sup toxicity indicated contribution of other virulence factors in addition to CLO.

**FIGURE 3:**
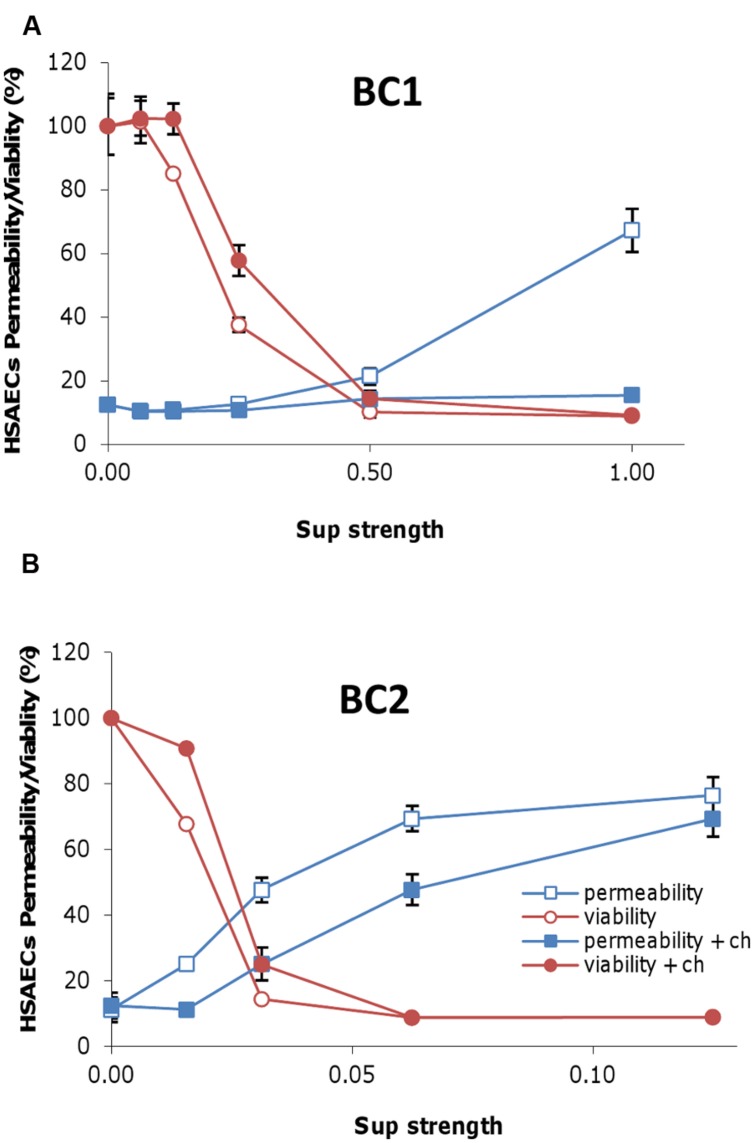
**The activity of Sups results in cell membrane damage of the exposed HSAECs.** Sups from microaerobic cultures of BC1 **(A)** and BC2 **(B)** in DMEM supplemented with 1 g L^-1^ of BSA were serially diluted with fresh medium and incubated with 10 μg mL^-1^ of cholesterol (ch) for 1 h at room temperature. Then 100 μl of Sups at the indicated dilutions were added to the monolayers of HSAECs and incubated for 20 min. The cell membrane permeability was assessed with the CytoTox-ONE Homogeneous Membrane Integrity kit (Promega) based on the release of LDH. Toxicity toward HSAECs was analyzed using resazurin. The viability and permeability corresponding assays were calculated as fluorescence relative to the untreated control or completely lysed cells, respectively.

To evaluate the effect of *B. cereus* PFTs on host cell membrane the amount of cell permeability in the Sup-treated HSAECs was measured using the Cytotox Homogenous Membrane Integrity Assay (Promega) which is based on the amount of released LDH. Both strains demonstrated a concentration-dependent increase in cell permeability, although the BC2 Sup required much lower concentration (higher dilution) to reach the effect comparable with BC1 Sup (**Figure 3**). The permeabilizing activity of BC1 Sup was almost completely inhibited in the presence of cholesterol (**Figure 3A**) while the BC2 Sup demonstrated only a partial cholesterol dependence (**Figure 3B**). The major drop in viability of exposed HSAECs for both strains took place at rather low levels of permeability when only a fraction of killed cells demonstrated LDH release. These results may be explained by a relatively high contribution of non-permeabilizing toxic factors. It is also likely that the membrane pores produced by the Sups were too small for the efficient translocation of LDH (which is a rather big tetrameric enzyme) but sufficient for the toxic effect of bacterial metabolic products (Walev et al., 2001).

### The MS Analysis of Size-Exclusion Column Filtrates of Microaerobic Sups Identifies Proteins Present in the Toxic Fractions

In order to further characterize virulence factors responsible for toxicity, microaerobic BC1 and BC2 cultures were grown, and the Sups from these cultures were concentrated and then fractionated using column chromatography. Sup fractions were added to HSAECs and the toxicity of each was measured using the redox-sensing resazurin dye. For both strains the main portion of toxicity was found in a peak corresponding to the proteins of approximate molecular mass of 50–60 kDa overlapping with the tail of the BSA peak (data not shown). LC-MS/MS analysis of the toxic fractions revealed a list of candidate toxic proteins (**Table 2**) along with the corresponding numbers of spectral counts (identified tryptic peptides) which can serve as a semi-quantitative measure of the protein abundance. Among the known pathogenic factors the fractions contained large amounts of collagenase, all three Hbl subunits, CLO and a hemolytic sphingomyelinase. No Nhe binding subunit, NheC, necessary for the enterotoxin activity was identified in either toxic fraction. It is plausible that the Nhe migrated through the column as dissociated individual subunits and therefore the NheC was separated from NheAB.

**Table 2:** Pathogenic factors identified in the toxic size-exclusion chromatography fractions of Sups.

Known virulence factor	Spectral count	
	BC1	BC2
Bacillolysin	1	0
Collagenase	22	25
Hemolysin BL binding component precursor	0	10
Hemolysin BL lytic component L1	0	12
Hemolysin BL lytic component L2	0	15
Non-hemolytic enterotoxin Nhe lytic component A	19	10
Non-hemolytic enterotoxin lytic Nhe component B	12	5
Cereolysin O precursor	11	5
Sphingomyelin phosphodiesterase	2	0

### Bacterial Propagation in Medium Supplemented with BSA Potentiates the Cytotoxicity of Sups

**FIGURE 4:**
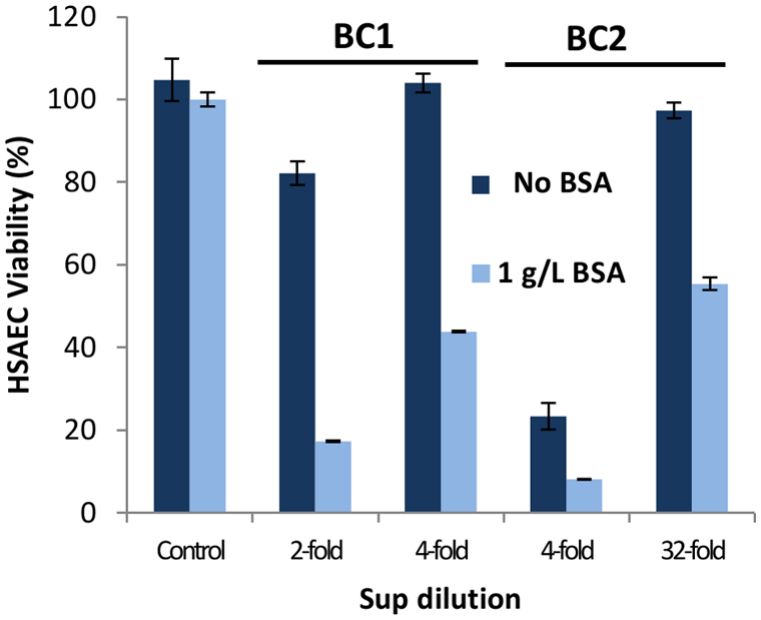
**The cultivation of *B. cereus* in medium supplemented with BSA enhances the cytotoxicity of Sups.** DMEM was supplemented with or without 1 g L^-1^ of BSA and Sups were generated from 20-h microaerobic cultures. Dilutions were prepared with the medium used for growing cultures and their toxicity to HSAECs assessed in triplicates using resazurin.

Previous research reported that supplementation of culture medium with BSA enhances the toxicity of the *Bacillus* species (Elleboudy et al., 2011; Popova et al., 2011; St John et al., 2013). To investigate this effect, cultures of BC1 and BC2 were grown in microaerobic conditions in DMEM supplemented with or without 1 g L^-1^ of BSA. **Figure 4** demonstrates BSA significantly increased the toxicity of bacterial Sups from both strains. The OD of these cultures indicated minor differences in growth that were not significant (0.72 OD^600^ in DMEM versus 0.80 OD^600^ in DMEM with BSA). Sups from BC1 cultures grown without BSA retained toxicity up to a fourfold dilution compared to the 32-fold dilution for the Sups from cultures grown in the presence of BSA. Reduction of the Sup pH from 6.9 to 5.4 was also noticed in the cultures grown in the presence BSA indicative of the stimulation of acidic anaerobic fermentation (Popova et al., 2011).

### BSA Prevents the Inactivation of the Reduced Isoform of CLO

**FIGURE 5:**
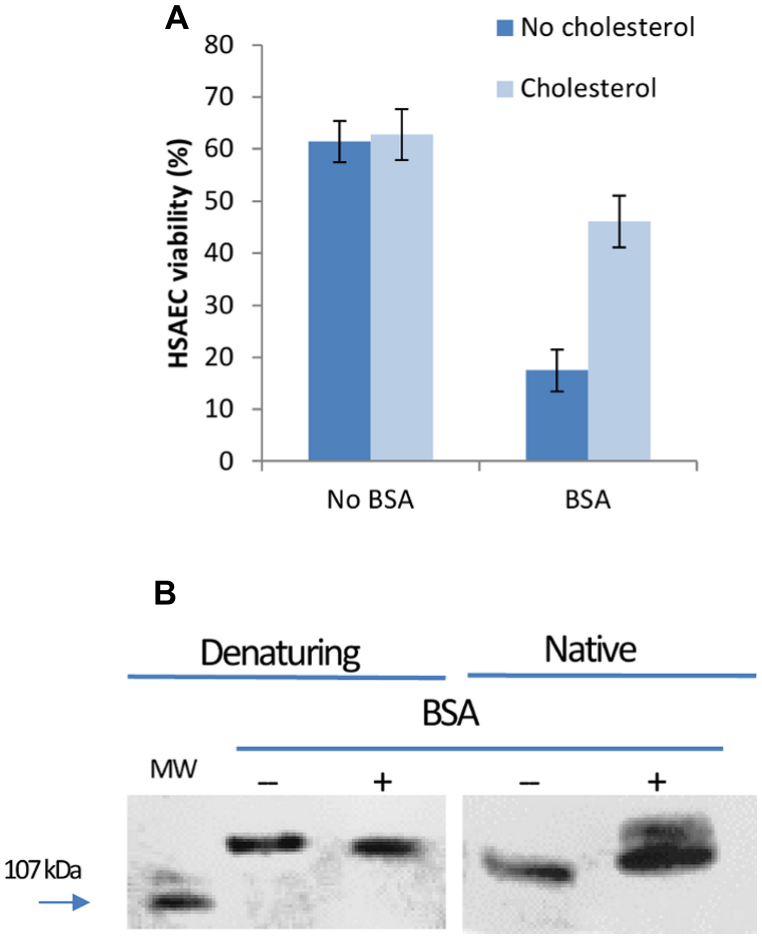
**CLO is inactivated in Sups concentrated in the absence of BSA. (A)** Sups from BC1 microaerobic cultures grown in DMEM with or without 1 g L^-1^ of BSA were filtered using Amicon centrifugal filters with a 3 kDa cut-off pore size. The retentates were diluted with corresponding medium to the original volume and assayed for CLO activity in triplicates after incubation with or without 10 μg mL^-1^ cholesterol for 1 h at room temperature. **(B)** Retentates from filtration were run on SDS-PAGE gels and transferred to nitrocellulose membranes. Membranes were blocked with 5% BSA in PBST and reacted with rabbit polyclonal anti-streptolysin O antibody followed by an anti-rabbit IgG, HRP-linked antibody. Blots were developed using SuperSignal West Dura Extended Duration Substrate (Thermo Scientific).

It was previously discovered that the effect of BSA may be relevant to stabilization of secreted CLO. Under certain conditions, CLO monomers spontaneously undergo pre-oligomerization which renders it unable to bind to cholesterol located on the cell membrane, resulting in reduced membrane permeabilization (Cowell et al., 1978; Gilbert, 2005). Additionally, CLO was reported to have two conformations depending on the oxidation or reduction of its cysteine residue. The active form contains a free sulfhydryl group and, if oxidized, results in protein inactivation. To determine the contribution of the above mechanisms to the CLO stabilization by BSA, BC1 cultures were grown in the presence or absence of BSA and concentrated by membrane filtration. To test if the concentration of Sups caused CLO inactivation the retentates were diluted with corresponding fresh media to the original volume and their toxicities were assessed after incubation in the presence or absence of cholesterol. After concentration, CLO only remained functional in Sups grown in the presence of BSA (**Figure 5A**).

The retentates were run on PAGE gels in native and denaturing conditions to analyze the protein mobility and pattern. Standard western blotting conditions were followed using antibody against highly homologous streptolysin O (Heffernan et al., 2007). On SDS-PAGE gel in the presence of DTT the protein demonstrated a single band corresponding to dimer with approximately 120 kDa in size (left panel in **Figure 5B**). The presence or absence of BSA appeared to have no effect on dimer formation in Sups. Right panel in **Figure 5B** demonstrates a native PAGE gel containing samples run without a reducing buffer. Sups from cultures grown in the presence of BSA had two bands of CLO, indicating different charged forms of the toxin. Previous research has identified the oxidized form of CLO migrates faster than its reduced form (Cowell et al., 1976). These results suggested BSA did not prevent CLO dimerization but instead protected an active conformation of CLO from being oxidized. BC2 strain demonstrated similar behavior; however, the amount of produced CLO was substantially reduced (data not shown).

### Metabolic Product Succinic Acid Acts as a Pathogenic Factor and is Enhanced by BSA

**FIGURE 6:**
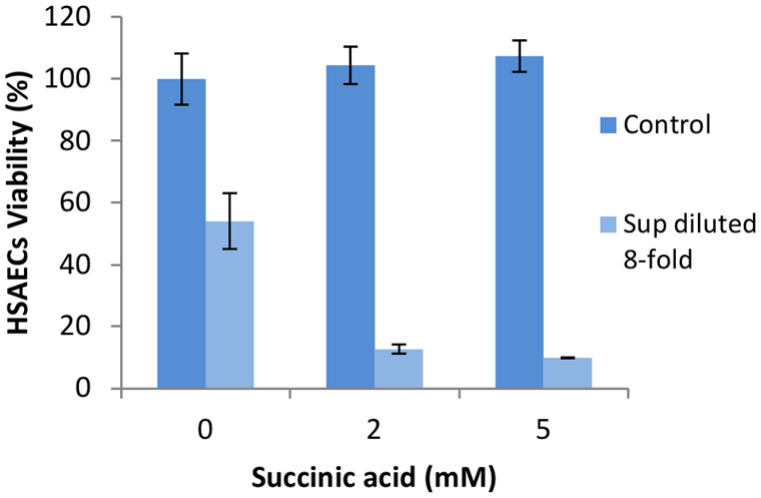
**Succinic acid (SA) increases the toxicity of microaerobic Sups grown in medium containing BSA.** Strain BC1 was inoculated into DMEM medium with 1 g L^-1^ of BSA and grown under microaerobic conditions for 20 h. Sups were diluted in DMEM titrated using HCl to the pH of Sups (5.4). After 20-min HSAECs exposure, Sups were removed and the cells were further incubated for 2 h at 37°C, 5% CO_2_ in DMEM supplemented with 0, 2, or 5 mM SA. After incubation, medium was removed and cell viability was assessed using resazurin. Controls included the same concentration of SA in the medium titrated to pH 5.4.

*Bacillus* species can undergo anaerobic fermentation that results in the generation and secretion of acidic metabolic products. Sups from BC1 grown in microaerobic conditions were titrated to pH 5.4, supplemented with SA and incubated with HSAECs. The pH value of 5.4 in Sups indicate SA which has pK_a_s of 4.2 and 5.6 could be a potential contributor in its partially protonated state. The concentration of SA in Sups was found to be 1.4 ± 0.1 mM (mean ± SD) regardless of BSA supplementation. To determine if succinate could potentiate *B. cereus* virulence, cells were briefly exposed to Sups which were then removed and replaced with medium containing different concentrations of SA. The supplementation with SA significantly enhanced the cytotoxicity of Sups (**Figure 6**).

### BSA Potentiates the Toxicity of *B. cereus* Mediated by Reactive Chemical Species

Previous research demonstrated that microaerobic cultures of *B. anthracis* generate NO and its derivatives such as NO_2_ and peroxynitrite. These substances enhance bacterial virulence through induction of mitochondrial stress in the exposed host cells (St John et al., 2013). The release of reactive species is detectable as a capacity of bacterial cultures to oxidize the dye AR as a test substrate (St John et al., 2013). Since *B. cereus* was also reported to produce peroxynitrite in microaerobic cultures and conditions of mild acid stress (Mols et al., 2010; Mols and Abee, 2011) we assessed the level of *B. cereus*-generated reactive substances using the AR test.

**FIGURE 7:**
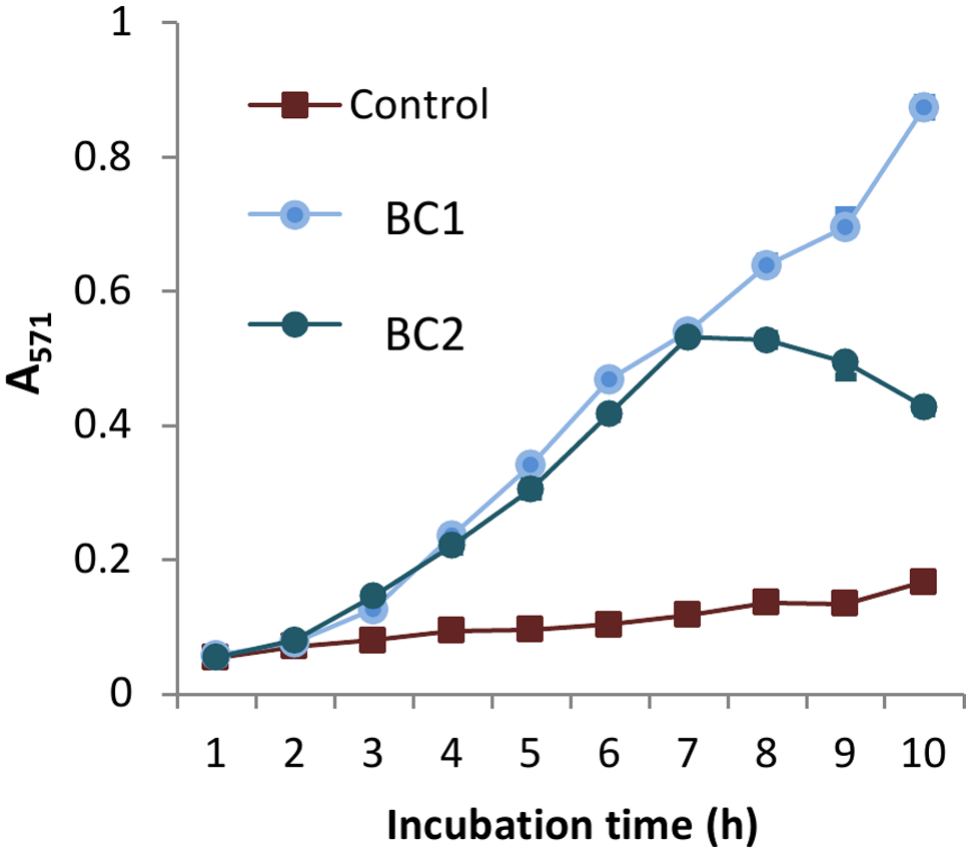
***B. cereus* produces reactive oxidizing species under microaerobic conditions.** BC1 and BC2 microaerobic cultures were grown in DMEM with 1 g L^-1^ of BSA, 0.1 mM of AR reagent, and 0.2 U L^-1^ of HRP using 24-well plates (1 mL per well). Sups were collected every hour and their absorbance was read in triplicates, *via* spectrophotometer at 571 nm. The reaction mixture including all components except the bacterial inoculum was used as a control.

AR is a colorless and non-fluorescent derivative of dihydroresorufin that generates a red derivative of resorufin when oxidized by hydrogen peroxide or peroxynitrite in the reaction catalyzed by HRP. Aliquots of bacterial cultures grown in the presence of AR and HRP were collected every hour and the color intensities of Sups were measured. A medium containing both AR and HRP reagents served as a control. **Figure 7** illustrates BC1 and BC2 microaerobic cultures generated significant amounts of oxidized AR. Upon a prolonged incubation with BC2 the intensity of released red color declined. This effect is typically associated with consumption of available oxygen (Towne et al., 2004). In addition, resorufin can be converted into a colorless product by peroxidase.

**FIGURE 8:**
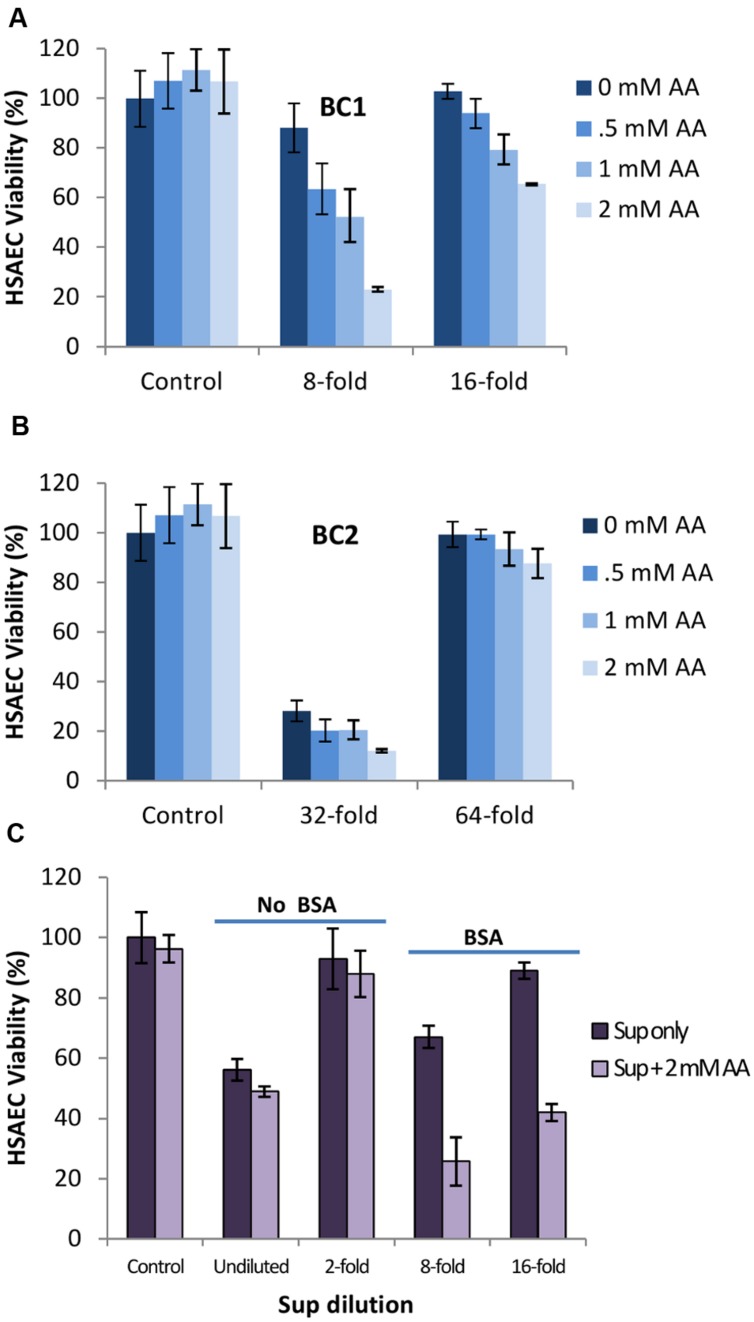
**Ascorbic acid (AA) potentiates the toxicity of Sups in BSA-dependent manner. (A,B)** Strains BC1 and BC2 were inoculated into DMEM medium with 1 g L^-1^ of BSA and grown under microaerobic conditions. Dilutions were made with medium titrated using HCl to the pH of grown cultures. Then, 100 mM stock of ascorbic acid in water was prepared and added to Sups at a concentration of 0, 0.5, 1, and 2 mM and incubated for 2 h at room temperature. The corresponding pH values were 5.42, 5.34, 5.28, and 5.08 in the case of BC1 and 5.35, 5.29, 5.11, and 4.96 in the case of BC2. HSAECs were exposed to the Sups for 20 min and viability was determined. **(C)** Strain BC1 was grown with and without BSA, and the effect of added AA was assessed as in **(A)** and **(B)**. Controls represent viability of mock-treated HSAECs.

To further elucidate the nature of the oxidizing substances, HSAECs were incubated with Sups supplemented with ascorbic acid (AA), a commonly used biologically relevant antioxidant (Bendich et al., 1986). For example, AA is effective in providing complete scavenging of NO-derived radicals from solution (Balavoine and Geletii, 1999). On the other hand, intracellular AA was reported to work as a pro-oxidant enhancing susceptibility of cells to the toxic effect of peroxynitrite in mitochondria (Guidarelli et al., 2001). We added AA to microaerobic Sups for 2 h at 0, 0.5, 1, and 2 mM concentrations and the viability of HSAECs was tested after 20-min exposure. Sups supplemented with ascorbate were considerably more toxic, and the BC1 Sup demonstrated the highest effect (**Figures 8A,B**). Control cells exposed to the AA-supplemented culture medium were non-toxic in spite of some acidification of the Sups by AA (**Figure 8** legend). The BC1 toxicity-enhancing effect of AA took place only in the presence of BSA (**Figure 8C**) in agreement with the previous studies implicating BSA in the capture and stabilization of reactive substances in Sups (St John et al., 2013).

## Discussion

The high toxicity of the *B. cereus* culture filtrates (Sups) in mice and cultured host cells was reported in several publications in the 1960s (Bonventre and Eckert, 1963a,b; Eckert and Bonventre, 1963; Bonventre, 1965). In comparison, Sups of *B. anthracis* cultures were almost completely non-toxic. This finding was rather unexpected, taking into account that commonly isolated *B. cereus* strains are not as virulent as *B. anthracis*. The conundrum remained unresolved as the attention of researchers shifted toward experiments with rats uniquely susceptible to anthrax Lethal Toxin. At that time a side-by-side comparison of *B. anthracis* and *B. cereus* required to characterize commonalities and differences between the pathogenic mechanisms employed by these microbes was complicated by the absence of genome sequence information. Current data demonstrate that many *B. cereus* strains possess a number of pathogenic factors with high cytotoxic potential and some of the recently discovered isolates display virulence *in vivo* comparable with *B. anthracis* (Hoffmaster et al., 2006; Oh et al., 2013). On the other hand, it is now understood that *B. anthracis* virulence seems to rely more on the immunomodulatory function of Lethal and Edema toxins rather than their direct cytotoxicity (Tournier et al., 2009).

Recent studies emphasized the importance of hypoxic conditions for the expression of *B. anthracis* pathogenic factors (Klichko et al., 2003; Popova et al., 2011, 2015; St John et al., 2013) thus providing rational explanation to the early reports which employed static (microaerobic) cultures (Thorne et al., 1960; Bonventre and Eckert, 1963a,b; Eckert and Bonventre, 1963). In the case of *B. cereus* there were conflicting studies regarding the influence of oxygen on enterotoxin production (Christiansson et al., 1989; Duport et al., 2004, 2006; Zigha et al., 2006; Van Der Voort and Abee, 2009). The regulation of these pathogenic factors has been shown to depend on the complex interaction of environmental parameters including bacterial density, oxygen availability, redox potential, temperature, glucose availability, and pH (Garcia-Arribas and Kramer, 1990; Glatz and Goepfert, 1976). The ResDE two-component system, as well as the anaerobic regulator Fnr, were found to exert major control on both fermentative growth and enterotoxin expression that function partially independently of the pleiotropic virulence gene regulator PlcR (Duport et al., 2006; Zigha et al., 2007).

In this study, we carried out a side-by-side analysis of cytotoxicity of two *B. cereus* strains in the conditions previously used to generate cytotoxic *B. anthracis* Sups due to expression of factors distinct from the Lethal and Edema toxins. Among the enterotoxin genes the strain BC1 (ATCC 11778) possesses the genes for CLO and Nhe while the strain BC2 has additional genes for Hbl, BceT, and CytK. We used HSAECs originating from the lung airways because the lung is uniquely susceptible to the Sups intravenously administered to mice and rats (in the cases of *B. cereus* and *B. anthracis*, respectively; Bonventre and Eckert, 1963a,b; Eckert and Bonventre, 1963). We found that for both strains a limited supply of oxygen in microaerobic (static) cultures delayed growth (**Figure 1**). However, both microaerobic and aerobic conditions resulted in a quick onset of HSAEC cytotoxicity within 2 to 4 h during a log-phase of growth. The effect of oxygen on the cytotoxic effect of Sups was strain-dependent: a microaerobic environment, compared to aeration, stimulated the toxicity of BC1, in contrast to BC2 which was more toxic upon aeration. However, irrespective of the influence of oxygen, the strain BC2 was substantially more toxic than BC1 judging by higher dilutions of the Sups required to reach a comparable effect on HSAECs (**Figure 2**). Based on the cholesterol inhibition of Sups’ toxicity we concluded that approximately half of the cytotoxic potential of both strains in microaerobic conditions (**Figure 2B**) depends on the activity of the CLO, assuming that other known toxins of *B. cereus* are not cholesterol-sensitive. In comparison, the effects of cholesterol on the toxicity of *B. cereus* and *B. anthracis* Sups are similar, but *B. anthracis* demonstrates markedly different time course of the exotoxin production. In microaerobic conditions *B. anthracis* displays no toxicity until a stationary phase, a finding that is consistent with the anaerobic control of ALO expression (Klichko et al., 2003). In line with this, aeration abrogates the activity of *B. anthracis* Sups which would otherwise require a 2-h cell exposure to elicit a toxic effect (Popova et al., 2011).

The activity of *B. cereus* PFTs in Sups was also demonstrated in experiments which evaluated the extent of cell membrane permeability based on the amount released LDH. Consistent with the toxic effect of small metabolic bacterial products, the large pores permeable to big LDH molecules were not necessary for the cell killing by Sups. The MS identification of the toxic products in the size exclusion column fractions confirmed the expression in BC2 Sups (but not BC1 Sups) of all three components of Hbl required for its activity (Sastalla et al., 2013). The Nhe and CLO were present in the Sups of both strains. The effect of cholesterol indicated CLO played a predominant role of membrane permeabilization by microaerobic BC1 Sups (**Figure 3A**). This finding is at odds with a conclusion on a major role of Nhe by Fagerlund et al. (2008). The discrepancy likely arises from the fact that the authors used aerobic cultures and therefore did not take into account the effect of toxic factors produced under anaerobic control.

A number of studies have reported BSA can significantly enhance the virulence of bacteria (Dubos, 1947; Liu, 1973; Elleboudy et al., 2011; St John et al., 2013; Kruczek et al., 2014). Research on *B. cereus* has found that medium supplemented with BSA increases the production of bacterial phospholipases (Elleboudy et al., 2011); however, we did not find the phospholipases in the toxic fractions of Sups (**Table 2**). Our experiments showed an additional mechanism in which BSA contributed to the maintenance of the active CLO conformation in Sups. Although CLO is believed to be primarily monomeric in solution (Cowell et al., 1978; Gilbert, 2005), our data indicate that in the Sups it was present in a dimeric form. BSA did not prevent CLO dimerization but instead protected the CLO dimer from being oxidized (**Figure 5**).

Additionally, serum albumin could concentrate NO and O_2_ in its hydrophobic core followed by the micellar catalysis of NO into N_2_O_3_ or NO_2_ which are more stable products (Nedospasov et al., 2000). Research further investigating this effect found NO may be responsible for downstream formation of toxic peroxynitrite and protein modifications negatively interfering with the host cell (St John et al., 2013). These authors found that the BSA supplementation into culture medium potentiated *B. anthracis* toxicity because of the BSA globule’s ability to concentrate and stabilize volatile NO bacterial products. We found that *B. cereus* displayed a metabolic toxicity similar to *B. anthracis* which was dependent on the presence of BSA. In line with the previous data, we confirmed that *B. cereus* generated oxidizing species with the properties of peroxynitrite (Mols and Abee, 2011; St John et al., 2013) which could be detected with the AR dye. The toxic potency of Sups was enhanced by SA, a metabolic by-product of bacteria under anaerobic conditions. In the case of *Bacteroides* species, SA is a known virulence product at pH of 5.5 but not at pH of 7.0 (Rotstein et al., 1987). The mechanism behind SA-induced toxicity involves reduction of intracellular pH which facilitates irreversible respiratory burst in the mitochondria. As it was suggested by Popova et al. (2011), in the presence of NO the burst may result in the formation of more toxic peroxynitrite.

To obtain additional evidence in favor of this mechanism we tested the effect of externally added AA which was reported to enhance the damaging effect of low concentrations of peroxynitrite (Guidarelli et al., 2001, 2014). AA, also known as vitamin C, is a very important water-soluble vitamin. Intracellular AA is involved in a large variety of biochemical reactions and generally displays antioxidant properties associated with prevention of the deleterious effects mediated by a large variety of reactive species. However, growing experimental evidence documents an unexpected ability of AA to enhance a peroxynitrite-dependent superoxide/H_2_O_2_ formation in the mitochondrial respiratory chain with the release of secondary species responsible for DNA damage and toxicity (Guidarelli et al., 2014). Indeed, addition of AA to the BC1 Sups reduced viability of HSAECs in the BSA-dependent manner (**Figure 8**) indicating participation of BSA in the chemical reactivity of Sups and supporting the previous data on the contribution of peroxynitrite (St John et al., 2013). However, AA did not increase the toxicity of BC2 Sups which therefore employ a mechanism different from BC1. Research in this direction is forthcoming.

In summary, our analysis demonstrates high potency of *B. cereus* strains BC1 (ATCC 11778) and BC2 (ATCC 14579) to produce secreted products with cytotoxic activity against lung epithelial cells in microaerobic and aerobic conditions strongly exceeding the previously reported activity of *B. anthracis* Sterne. In the case of both strains the PFTs contribute substantially to the mechanism involving permeabilization of the target cell membrane combined with the effect of acidic metabolic products and the host serum albumin in microaerobic environment. With regard to our findings, we hypothesize that the tested strains of *B. cereus* and *B. anthracis* evolved to rely on different pathogenic strategies in which the former one emphasizes a direct cytotoxicity while the latter one is much less cytotoxic but strongly immunomodulating. Our results were obtained with only one cell type and therefore do not reflect a potential variety of cell-specific features of *B. cereus* pathogenic factors. Nevertheless, the behavior of epithelial cells well known to be highly susceptible to PFTs is relevant to different biological scenarios. Further characterization of *B. cereus* strains in different environmental conditions is required to fully understand pathogenic mechanisms employed by this microbe.

## Author Contributions

Contributed to conception and design: SP and KK. Contributed to acquisition, analysis, and interpretation of data: KK, TP, and AT. Drafted and/or revised the article: KK, SP, TP, and AT.

## Conflict of Interest Statement

The authors declare that the research was conducted in the absence of any commercial or financial relationships that could be construed as a potential conflict of interest.
